# Fine Mapping of *Ur-3*, a Historically Important Rust Resistance Locus in Common Bean

**DOI:** 10.1534/g3.116.036061

**Published:** 2016-12-27

**Authors:** Oscar P. Hurtado-Gonzales, Giseli Valentini, Thiago A. S. Gilio, Alexandre M. Martins, Qijian Song, Marcial A. Pastor-Corrales

**Affiliations:** * Soybean Genomics and Improvement Laboratory, United States Department of Agriculture-Agricultural Research Service, Beltsville Agricultural Research Center-West, Maryland 20705; † Departamento de Agronomia, Universidade Estadual de Maringá, PR 87020900, Brazil; ‡ Coordenação de Tecnologia em Educacao a Distancia, Coordenação de Aperfeiçoamento de Pessoal de Nivel Superior, Quadra St. Bancário Norte, Brasília, DF 70040020, Brazil

**Keywords:** *Phaseolus vulgaris*, *Uromyces appendiculatus*, fine mapping, rust resistance gene, KASP marker

## Abstract

Bean rust, caused by *Uromyces appendiculatus*, is a devastating disease of common bean (*Phaseolus vulgaris*) in the Americas and Africa. The historically important *Ur-3* gene confers resistance to many races of the highly variable bean rust pathogen that overcome other rust resistance genes. Existing molecular markers tagging *Ur-3* for use in marker-assisted selection produce false results. Here, we describe the fine mapping of the *Ur-3* locus for the development of highly accurate markers linked to *Ur-3*. An F_2_ population from the cross Pinto 114 (susceptible) × Aurora (resistant with *Ur-3*) was evaluated for its reaction to four different races of *U. appendiculatus*. A bulked segregant analysis using the SNP chip BARCBEAN6K_3 placed the approximate location of *Ur-3* in the lower arm of chromosome Pv11. Specific SSR and SNP markers and haplotype analysis of 18 sequenced bean varieties positioned *Ur-3* in a 46.5 kb genomic region from 46.96 to 47.01 Mb on Pv11. We discovered in this region the SS68 KASP marker that was tightly linked to *Ur-3*. Validation of SS68 on a panel of 130 diverse common bean cultivars containing all known rust resistance genes revealed that SS68 was highly accurate and produced no false results. The SS68 marker will be of great value in pyramiding *Ur-3* with other rust resistance genes. It will also significantly reduce time and labor associated with the current phenotypic detection of *Ur-3*. This is the first utilization of fine mapping to discover markers linked to rust resistance in common bean.

The common bean (*Phaseolus vulgaris* L.) includes dry and snap beans. The dry edible bean is the most important pulse in the diet of humans throughout the world, especially in Latin America and Africa, where dry beans are the main daily source of protein, complex carbohydrates, fiber, and micronutrients, particularly for the poorest populations (Broughton *et al.* 2003).

A myriad of biotic and abiotic factors constrain common bean production in the world. Among these, bean rust is a devastating disease that results in significant loss of seed yield in dry beans and pod quality in snap beans (Stavely and Pastor-Corrales 1989; Liebenberg and Pretorius 2010).

The bean rust disease is caused by the biotrophic basidiomycete fungus *Uromyces appendiculatus*, an obligate parasite of common bean. This pathogen has a complex life cycle with five distinct spore stages and three different nuclear conditions, which are indicative of the capacity of this pathogen for genetic recombination (Groth and Mogen 1978; McMillan *et al.* 2003). Many published reports reveal the rich virulence diversity of *U. appendiculatus*, with scores of races (virulence phenotypes) identified around the world (Groth and Roelfs 1982; Mmbaga and Stavely 1988; Stavely and Pastor-Corrales 1989; Liebenberg 2003; Araya *et al.* 2004; Arunga *et al.* 2012; Acevedo *et al.* 2012). More than 90 races of *U. appendiculatus* from the United States, Africa, Asia, and other countries of the Americas have been characterized and maintained by the United States Department of Agriculture-Agricultural Research Service Bean Project at the Beltsville Agricultural Research Center (Stavely 1984; Mmbaga and Stavely 1988; Stavely *et al.* 1989; Pastor-Corrales 2001).

Genetic resistance is the most cost-effective strategy to manage bean rust disease. Rust resistance in common bean is conditioned by single and dominant genes identified by the *Ur*- symbol (Kelly *et al.* 1996). To date, 10 genes have been named and tagged, mostly with RAPD or SCAR molecular markers (Miklas *et al.* 2002). Five genes (*Ur-3*, *Ur-5*, *Ur-7*, *Ur-11*, and *Ur-14*) belong to the Middle American gene pool, while five genes (*Ur-4*, *Ur-6*, *Ur-9*, *Ur-12*, and *Ur-13*) belong to the Andean gene pool (Augustin *et al.* 1972; Ballantyne 1978; Stavely 1984, 1990; Grafton *et al.* 1985; Finke *et al.* 1986; Jung *et al.* 1998; Liebenberg and Pretorius 2004; Souza *et al.* 2011).

The *Ur-3* gene present in the Middle American white-seeded common bean, Aurora, was reported by Ballantyne (1978). Since then, this gene has been used extensively as the source of rust resistance in a large number of dry bean cultivars from various market classes of the United States, as well as in fresh market and processing snap beans (Kelly *et al.* 1994; Stavely *et al.* 1997; Pastor-Corrales *et al.* 2007; Urrea *et al.* 2009; Osorno *et al.* 2010; Brick *et al.* 2011; Beaver *et al.* 2015). *Ur-3* has also been used as a source of rust resistance in dry bean cultivars of South Africa (Liebenberg *et al.* 2005). In addition, *Ur-3* has been the subject of different studies, including genetics (Grafton *et al.* 1985; Kalavacharla *et al.* 2000), molecular markers, and gene tagging (Haley *et al.* 1994). The *Ur-3* is also present in Middle American cultivars Mexico 235, Ecuador 299, NEP 2, and 51052, in addition to other undefined rust resistance genes (Stavely *et al.* 1989; Miklas *et al.* 2000; Hurtado-Gonzales *et al.* 2016).

The *Ur-3* gene confers resistance to 55 of 94 races of the bean rust pathogen maintained at Beltsville, MD (Pastor-Corrales *et al.* 2001). More importantly, *Ur-3* confers resistance to many races that overcome the resistance of all other named rust resistance genes in common bean. For example, the *Ur-3* gene confers resistance to race 22-52 (previously known as race 108), the only race known to overcome the broad-spectrum resistance of the *Ur-11* gene present in PI 181996 and PI 190078, and of the *Ur-14* gene present in Ouro Negro (Stavely 1998; Alzate-Marin *et al.* 2004). The name of race 108 and of six other races (41, 47, 49, 53, 67, and 84) used in this study, was changed after these races were phenotyped on a new set of bean rust differential cultivars adopted for the characterization of races of *U. appendiculatus* and a binary system to name these races (Steadman *et al.* 2002; Pastor-Corrales and Aime 2004). The new and old names (in parentheses) of the races used in this study are: 15-1 (41), 15-3 (47), 22-6 (49), 31-1 (53), 31-22 (67), 37-1 (84), and 22-52 (108).

The *Ur-3* gene also complements the broad-spectrum rust resistance in accessions PI 151385, PI 151388, PI 151395, and PI 151396, which are also only susceptible to race 22-52. Similarly, *Ur-3* confers resistance to race 37-1, the only known race that overcomes the rust resistance in PI 260418 (Pastor-Corrales 2005). In addition, *Ur-3* confers resistance to many races that overcome the *Ur-4*, *Ur-5*, *Ur-6*, *Ur-7*, *Ur-9*, *Ur-12*, and *Ur-13* genes. Although *Ur-3* is not resistant to all races of Mesoamerican origin, this gene confers resistance to most races of *U. appendiculatus* of Andean origin; that is, races isolated from common beans of the Andean gene pool. Thus, *Ur-3* is a critical component of gene pyramiding of common bean cultivars with broad resistance to rust. The information above provides strong evidence of the historical importance and current relevance of *Ur-3* for breeding dry and snap beans with broad and durable resistance to rust in the United States and other nations (Stavely 2000; Pastor-Corrales *et al.* 2001).

The resistant reaction of *Ur-3* gene to *U. appendiculatus* is initially characterized by the production of small water-soaked chlorotic spots that subsequently become, in ∼48 hr, well-defined necrotic spots without sporulation. This resistant phenotype is classified as grade 2, 2+ and it is known as the hypersensitive reaction (HR) in the bean rust grading scale (Stavely *et al.* 1989; Stavely 1998).

The *Ur-3* gene has been mapped on chromosome Pv11 of the common bean genome (Stavely 1998; Miklas *et al.* 2002). Inheritance of resistance and phenotypic data revealed that the *Ur-3* gene was very closely linked to *Ur-11* on the terminal position of chromosome Pv11 (Kelly *et al.* 1996). The close proximity between these two genes led to the naming of the rust resistance gene in PI 181996 as *Ur-3^2^* (Kelly *et al.* 1996). However, later reports demonstrated the independence of *Ur-3* and *Ur-3^2^* and revealed that these two genes were linked in repulsion and different from each other (Stavely 1998). Thus, *Ur-3^2^* was renamed *Ur-11* (Stavely 1998). The close proximity of *Ur-3*–*Ur-11* may be one of the main reasons why it has been difficult to find DNA markers that are specific for the *Ur-3* gene. There are other named rust resistance genes (*Ur-6* and *Ur-7*) on Pv11, as well as two other unnamed genes (*Ur-Dorado 53* and *Ur-BAC 6*), although these genes are not as tightly linked to *Ur-3* as *Ur-11* (Miklas *et al.* 2002; Kelly *et al.* 2003).

Four specific races of the bean rust pathogen have been reported as phenotypic markers that effectively identify rust resistance genes; race 31-1 identifies *Ur-3*, race 22-6 recognizes *Ur-4*, race 15-3 identifies *Ur-6*, and race 31-22 recognizes *Ur-11* (Stavely 2000; Pastor-Corrales and Stavely 2002). These races identify the presence of these genes alone or in combination with other rust resistance genes. However, the phenotypic identification of these rust resistance genes is laborious, time consuming, and currently only performed at the Bean Project at Beltsville, MD. Moreover, the detection of multiple rust resistance genes in common bean using phenotypic markers is also often complicated by the presence of epistasis between rust resistance genes (Miklas *et al.* 1993; Pastor-Corrales and Stavely 2002). Furthermore, the current molecular markers (mostly RAPD and SCAR markers) linked to rust resistance genes in common bean that were published almost two decades ago, yield false positive and false negative results, as indicated by the authors that reported the currently available RAPD (OK14_620_) and SCAR (SK14) markers linked to the *Ur-3* locus (Haley *et al.* 1994; Nemchinova and Stavely 1998).

Several factors contributed to the false positive and false negative results when using the current molecular markers. Among these factors is the weak linkage of some molecular markers with the gene of interest. For instance, the RAPD marker (OK14_620_) tagging *Ur-3*, was reported to be positioned 2.23 cM from this gene (Haley *et al.* 1994). Another constraint was the close proximity of rust resistance genes, as is the case with the *Ur-3* and *Ur-11* genes. Additionally, the lack of a reference genome for common bean hindered the development of highly specific, tightly linked DNA markers. The publication of the common bean reference genome in 2014 (Schmutz *et al.* 2014), along with the development of high-throughput genotyping technologies for common bean, are making possible the identification of more effective molecular markers.

Although the *Ur-3* is a very important rust resistance gene in common bean, to date there is not a reliable molecular marker tagging *Ur-3*. Thus, to improve the durability of common bean cultivars to the highly variable bean rust pathogen, *Ur-3* cannot be combined with other rust resistance genes using marker-assisted selection. As indicated earlier, at present, pyramiding *Ur-3* with other rust resistance genes is only feasible using specific races of the rust pathogen, an activity that is reliable but highly laborious and time consuming. The objective of this study was to develop highly specific, tightly linked, effective molecular markers for the detection of the historically important and widely used *Ur-3* rust resistance gene, either alone or in combination with other rust resistance genes of common bean.

## Materials and Methods

### Population development and phenotypic evaluation of the bean rust disease

A total of 129 F_2_ plants were derived from the cross Pinto 114 × Aurora. Both are dry beans of the Middle American pool of common bean, where Pinto 114 was the susceptible parent and Aurora was the resistant parent containing the *Ur-3* gene. The following cultivars with known rust resistance genes were included in the inoculation as internal controls of successful rust inoculation: Early Gallatin (*Ur-4*), Golden Gate Wax (*Ur-6*), and PI 181996 (*Ur-11*) (Table 1). All F_2_ plants, parents, and control cultivars were grown in 12.7-cm diameter pots containing two plants per pot. The primary (unifoliate) leaves of bean plants were inoculated ∼7 d after seeding, when the primary leaves were ∼35–65% expanded (Stavely 1984). To prepare the rust inocula, suspensions of frozen urediniospores of various races of *U. appendiculatus* were placed in a 25-ml solution of cold tap water and 0.01% Tween 20 in a 250-ml Erlenmeyer flask. The spore solutions were prepared with a concentration of 2 × 10^4^ urediniospores per ml^−1^. All 129 F_2_ plants and the control cultivars were inoculated with races 15-1, 31-1, 37-1, and 22-52 of *U. appendiculatus*. Races 15-1, 31-1, 37-1, and 22-52 elicited the same resistance (HR) reaction on plants with *Ur-3*, as shown in Supplemental Material, Table S1. However, these races elicited a different type of reaction on PI 181996 (the control cultivar with *Ur-11*) and on cultivars with other rust resistance genes. Thus, one important reason for using four races to phenotype the F_2_ population was to unequivocally ensure the phenotype of each F_2_ plant, the parents, and of the control cultivars, which included plants with *Ur-4*, *Ur-6*, *Ur-11*, and other rust resistance genes. The F_2_ plants were inoculated using a cotton swab to apply the spore solution of each of the races on the abaxial side of the primary leaves. After inoculation, the plants were transferred to a mist chamber (20 ± 1° and relative humidity >95%) for 18 hr, under darkness. After this period, the plants were transferred to the greenhouse. Visible rust symptoms were observed on susceptible plants ∼10–12 d after inoculation (dai).

**Table 1:** Reaction of the common bean cultivars used in this study to races 15-1 (41), 31-1 (53), 37-1 (84), and 22-52 (108) of *Uromyces appendiculatus*, the causal agent of the bean rust disease

Cultivar	*Ur* Gene	Reaction to Races of *Uromyces appendiculatus*			
		15-1	31-1	37-1	22-52
Pinto 114	*–*	5, 4	5, 4	5, 4	5, 4, 6
Aurora	*Ur-3*	2	2+	2+	2,2+
Early Gallatin	*Ur-4*	4, 5	4, 5	4, 5	2+
Golden Gate Wax	*Ur-6*	3, f2	4, 5	3, f2	4, 5
PI 181996	*Ur-11*	f2	f2	f2	5,6

Standard bean rust grading scale: 1 = no visible symptoms; 2,2+ = necrotic spots without sporulation; f2 = faint and tiny chlorotic spots; 3 = tiny uredinia (sporulating pustules) <0.3 mm in diameter; 4 = uredinia, 0.3–0.5 mm in diameter (large sporulating pustules); 5 = large uredinia, 0.5–0.8 mm in diameter, 6 = very large uredinia, >0.8 mm in diameter. Reactions 2, 3, and f2 are considered **r**esistant. Reactions 4, 5, and 6 are considered **s**usceptible.

The F_2_ population and parents were evaluated for their rust phenotype ∼12–14 dai using a 1–6 scale (Stavely and Pastor-Corrales 1989), scored as follows: 1 = no visible rust symptoms; 2 = necrotic or chlorotic spots without sporulation, <0.3 mm in diameter (HR); 2+ = necrotic spots without sporulation, 0.3–1.0 mm in diameter; 2++ = necrotic spots without sporulation, 1.0–3.0 mm in diameter; 2+++ = necrotic spots >3.0 mm in diameter; 3 = uredinia <0.3 mm in diameter (tiny sporulating pustules); 4 = uredinia 0.3–0.5 mm in diameter (large sporulating pustules); 5 = uredinia 0.5–0.8 mm in diameter (large sporulating pustules); and 6 = uredinia >0.8 mm in diameter (very large sporulating pustules). Plants with grades 2 and 3 were classified as resistant, whereas those with rust grades of 4, 5, or 6 were classified as susceptible. Thereafter, the F_2_ plants were maintained in the greenhouse to produce F_2:3_ families by self-fertilization. A total of 281 F_3_ plants from 12 selected F_2:3_ families were inoculated with race 31-1 of *U. appendiculatus*. These families were inoculated using an Air Brush-Depot compressor, model TC-20, and an Iwata Airbrush, Revolution BCR, by applying the spore solution (concentration of 2 × 10^4^ per ml^−1^) of race 31-1 on the abaxial side of the leaves. After spraying, plants were treated similarly to the F_2_ plants, as described above. The reaction (rust phenotype) of the differential bean cultivars to all races of *U. appendiculatus* used in this study is presented in Table S1.

### Bulk segregant analysis and single nucleotide polymorphism assay

Newly emerged trifoliate leaves from each of the F_2_ plants were collected and total genomic DNA was isolated using DNeasy 96 Plant Kit (Qiagen, Valencia, CA) according to manufacturer’s instructions. Based on the rust reaction of each of the F_2_ plants, three susceptible (rr) bulks were prepared. Each bulk consisted of DNA from eight different F_2_ susceptible plants. Bulks of resistant F_2_ plants were not prepared to avoid the inclusion of heterozygous-resistant (Rr) plants. These bulks were used for bulk segregant analysis (BSA) for identification of markers potentially associated with the *Ur-3* gene (Michelmore *et al.* 1991). The DNA from susceptible bulks and two samples from each parent were analyzed with 5398 single nucleotide polymorphism (SNP) markers on the Illumina BeadChip BARCBEAN6K_3, following the Infinium HD Assay Ultra Protocol (Illumina, Inc., San Diego, CA). The results obtained on the BeadChip were visualized by fluorescence intensity using the Illumina BeadArray Reader and alleles were called using Illumina GenomeStudio V2011.1 (Illumina, Inc.). Allele calls were visually inspected and errors in allele calling were corrected manually. SNPs were considered to be associated with the *Ur-3* locus when they were polymorphic between the Pinto 114 (susceptible) and Aurora (resistant) parents and the three susceptible bulks were homozygous and clustered tightly with the susceptible parent (Pinto 114).

### Developing and evaluating simple sequence repeat markers linked to Ur-3

The sequence fragments containing SNPs associated with the *Ur-3* locus were aligned to the common bean reference genome using Standalone Megablast (Morgulis *et al.* 2008) to identify the scaffolds in the reference genome. Scaffolds were downloaded from Phytozome (https://phytozome.jgi.doe.gov/pz/portal.html), DOE, JGI (Department of Energy, Joint Genome Institute). The scaffolds were screened for the presence of simple sequence repeat (SSR) markers. Procedures for SSR identification, SSR screening, and primer design were previously described by Song *et al.* (2010).

The polymorphism and quality of the SSR markers were first tested using DNA from the Pinto 114 (susceptible) and Aurora (resistant) parents. Polymorphic SSR markers between Pinto and Aurora were then used to analyze the DNA of the F_2_ population from the Pinto 114 × Aurora cross. Polymerase chain reaction (PCR) was performed with 5 ng of genomic DNA, 0.25 μM of forward and reverse primers, 1× PCR buffer [200 mM Tris-HCl (pH 8.0), 500 mM KCl, 2 mM each dNTP, 10% glycerol, 15 mM MgCl_2_, 20 ng/µl of single-stranded binding protein], and 0.1 unit of Taq DNA polymerase. The PCR thermocycling parameters were 3 min at 92° and 38 cycles of 50 sec at 90°, 45 sec at 58°, and 45 sec at 72°, followed by a 5 min extension at 72° and hold at 10°. PCR products were resolved on 2–3% agarose gels (Agarose SFR; Amresco, Dallas, TX) prepared with TBE 1× buffer (Tris-borate-EDTA) and stained with 1 μg/ml^−1^ ethidium bromide.

### Developing and testing Kompetitive Allele Specific PCR markers

A subset of SNPs positively associated with *Ur-3* found using BSA were selected for genotyping the F_2_ population from Pinto 114 × Aurora using Kompetitive Allele Specific PCR (KASP) assays. Additional SNPs used for KASP genotyping were retrieved from SNP chip tables found in Song *et al.* (2015). KASP primers were designed using the PrimerExpress software and KASP reactions were conducted following the manufacturer’s instructions. The 10 μl reaction contained 5 μl of 2× premade KASP master mix (LGC, Middlesex, UK), 0.14 μl of primers mix (Sigma-Aldrich, St. Louis, MO), and 20–40 ng of genomic DNA. PCR parameters were as described by LGC, on standard thermocycling machines, using white semiskirted polypropylene 0.2 ml 96-well PCR plates (USA Scientific), and sealed with MicrosealB (Bio-Rad, Hercules, CA). After PCR amplification was completed, PCR plates were scanned using the Mx3000P qPCR machine (Agilent, St. Clara, CA) and allele calls for each genotype were obtained using the MxPro software (Agilent) or the Klustercaller software (LGC).

### Construction of genetic linkage map around the Ur-3 locus

The genetic distance between the SSRs, KASPs, and the *Ur-3* locus in the F_2_ population (129 plants) was estimated using the software JoinMap 4.0 (Van Ooijen 2006). Default settings of a Regression Mapping algorithm based on Kosambi map function were attributed to define linkage order and distances in centimorgans (cM). A minimum likelihood of odds (LOD) ≥3.0 and a maximum distance of ≤50 cM were used to test linkages among markers.

### Fine mapping of the Ur-3 locus in F_3_ plants using KASP markers

F_3_ families were selected based on the recombination between *Ur-3* and the SSRs and KASPs molecular markers found in the F_2_ population. A total of 10 F_3_ families were selected for screening with KASP markers SS4 and SS6 flanking the *Ur-3* locus. One homozygous-resistant family and one susceptible family were evaluated as internal controls. The number of plants per family varied from 22 to 32, according to the availability of seeds. A total of 281 F_3_ plants were inoculated with race 31-1 of *U. appendiculatus*, as described in *Materials and Methods*. DNA from the F_3_ plants was isolated according to Lamour and Finley (2006) and were genotyped with KASP markers SS4 and SS6. F_3_ plants showing recombination between markers SS4 and SS6 were selected for additional genotyping with newly designed KASP markers in order to narrow the genomic region containing the *Ur-3* locus.

### Haplotype analysis of the Ur-3 locus

Haplotype analysis was performed in the genomic region flanked by the SS4 and SS6 KASP markers. These two markers flanked a region of 470,487 bp on Pv11, from 46,613,419 to 47,083,906 bp. Eighteen diverse bean varieties, including C 20, Matterhorn, Stampede, T-39, Sierra, Red Hawk, Jalo EEP 558, Michelite, UC White, Kardinal, Laker, Cornell 49242, BAT 93, Buckskin, Fiero, Lark, UI 906, and CELRK, were sequenced by Song *et al.* (2015) and used for the haplotype analysis. These lines were also inoculated with races 22-6, 31-1, 31-22, and 22-52 of *U. appendiculatus*. The four races were used to identify the presence of certain rust resistance genes in these cultivars; races 31-1 and 22-52 to identify the presence of *Ur-3*, race 31-22 to identify *Ur-11*, and race 22-6 to identify *Ur-4*. Cultivars with HR to races 31-1 and 22-52 had the *Ur-3* gene (Table S1). The sequence variants in the targeted genomic region of the 18 varieties and their phenotypes were used to identify haplotypes associated with resistance and susceptibility to race 31-1. All SNPs identified between KASP markers SS4 and SS6 were handled using Microsoft Excel and haplotypes were identified by visual inspection. At least two KASP markers were designed for each of the observed haplotypes. Whenever feasible, SNP markers were located every 10 kb across the 470,487 bp genomic region. When KASP markers were polymorphic between the Pinto 114 (*ur-3*) and Aurora (*Ur-3*) parents, they were used to genotype F_3_ plants with recombination between the markers SS4 and SS6.

### Validation of the markers linked to the Ur-3 locus

A panel of 130 diverse bean cultivars that included all rust resistance genes in common bean were genotyped using KASP markers tightly linked with *Ur-3*. This was performed with the purpose of generating accurate *Ur-3* markers useful in marker-assisted selection. In this panel, some cultivars had the *Ur-3* gene alone, other cultivars had *Ur-3* combined with other rust resistance genes, while others did not have any reported rust resistance genes. The cultivars in the panel were phenotyped before or during this study with multiple races of the bean rust pathogen, including race 31-1, the phenotypic marker for the *Ur-3* gene.

### Data availability

All data described in this manuscript related to bean rust phenotypes, Pinto 114 × Aurora F_2_ genetic map, F_3_ fine-mapping population, and haplotype analysis are available in Table S1, Table S2, Table S3, Table S4, Table S5, Table S6, and Table S7.

## Results

### Inheritance of rust resistance in common bean Aurora

A total of 129 F_2_ plants from the Pinto 114 × Aurora cross were evaluated for their reaction to races 15-1, 31-1, 37-1, and 22-52 of *U. appendiculatus* (Table S2). Aurora was resistant to all four races and exhibited the same type of reaction that was characterized by necrotic spots without sporulation (grades 2, 2+). Pinto 114 was susceptible to the same four races, with a reaction characterized by large uredinia (grades 4, 5, and 6). Based on the reaction to all four races, the inheritance of rust resistance study of the 129 F_2_ plants exhibited a segregation equal to 101 resistant and 28 susceptible plants, fitting a ratio of 3 resistant to 1 susceptible (χ^2^ = 0.747, *P* value = 0.38), confirming that the rust resistance in Aurora was conferred by the single and dominant *Ur-3* gene (Table S2).

### BSA and SNP genotyping using BARCBEAN6K_3 BeadChip

Based on the BSA, 28 SNPs were associated with *Ur-3* (Table 2). The alleles of these SNPs could separate the susceptible Pinto 114 and the three susceptible bulks from the resistant Aurora parent. According to the genetic linkage map created by Song *et al.* (2015), these 28 SNPs were distributed from 72.3 to 76.2 cM on the lower end of the common bean chromosome Pv11. The physical location of the associated 28 SNPs was between 46,437,627 bp (ss715647455) and 48,784,158 bp (ss715641910), a region spanning a total of 2.1 Mbp (Table 2).

**Table 2:** Positive single nucleotide polymorphism (SNP) markers associated with the *Ur-3* locus in the common bean linkage group Pv11

NCBI ssID	BARCBEAN6K_3 SNP ID	Physical Position Pvul V1.0 (bp)	SR F_2_ Linkage Map Position (cM)*^a^*
ss715647455	sc00206ln407767_400194_T_G_143587659	46,437,627	72.337
ss715639564	sc00206ln407767_348254_C_T_143535719	46,490,018	—
ss715647451	sc00206ln407767_168453_G_A_143355918	46,667,862	72.512
ss715647773	sc00273ln341540_84911_C_A_168094735	46,939,681	73.921
ss715647765	sc00273ln341540_130419_A_G_168140243	46,982,186	73.921
ss715647770	sc00273ln341540_233731_C_A_168243555	47,083,906	—
ss715640322	sc00733ln158243_112892_C_T_274461500	47,289,130	—
ss715649250	sc00733ln158243_110962_C_T_274459570	47,291,059	—
ss715649254	sc00733ln158243_81656_T_G_274430264	47,320,724	74.735
ss715649249	sc00733ln158243_10639_C_T_274359247	47,390,755	75.07
ss715649251	sc00733ln158243_2983_T_C_274351591	47,398,413	75.07
ss715649719	sc00992ln119304_109010_C_T_310044208	47,431,965	75.07
ss715648098	sc00346ln293441_287396_T_C_191559002	47,746,437	75.684
ss715648096	sc00346ln293441_269534_C_T_191541140	47,768,651	75.07
ss715648093	sc00346ln293441_239954_T_C_191511560	47,800,050	75.07
ss715649910	sc01089ln106922_89228_T_C_320994162	48,163,156	75.222
ss715640836	sc01089ln106922_30683_A_G_320935617	48,221,254	75.222
ss715648349	sc00418ln255472_36179_T_C_211051790	48,547,014	76.258
ss715648350	sc00418ln255472_49970_A_G_211065581	48,560,374	76.258
ss715648351	sc00418ln255472_79205_G_A_211094816	48,588,580	—
ss715648352	sc00418ln255472_96331_T_G_211111942	48,605,710	—
ss715648342	sc00418ln255472_105778_T_G_211121389	48,614,962	—
ss715648343	sc00418ln255472_112102_G_T_211127713	48,621,286	76.258
ss715648344	sc00418ln255472_133412_T_C_211149023	48,640,040	—
ss715648345	sc00418ln255472_153753_G_A_211169364	48,660,384	—
ss715648346	sc00418ln255472_173904_T_C_211189515	48,680,290	76.258
ss715650748	sc01832ln56221_26620_C_T_378717205	48,780,038	76.258
ss715641910	sc01832ln56221_30739_G_A_378721324	48,784,158	76.258

These markers, identified using bulk segregant analysis and the BARCBEAN6k_3 BeadChip, were polymorphic between the resistant Aurora (*Ur-3*) and the susceptible Pinto (*ur-3*) parents and were associated with the susceptible (*ur-3*) bulks.

a Genetic position based on Song *et al.* (2015) genetic map.

### Mapping of the Ur-3 gene

**Figure 1:**
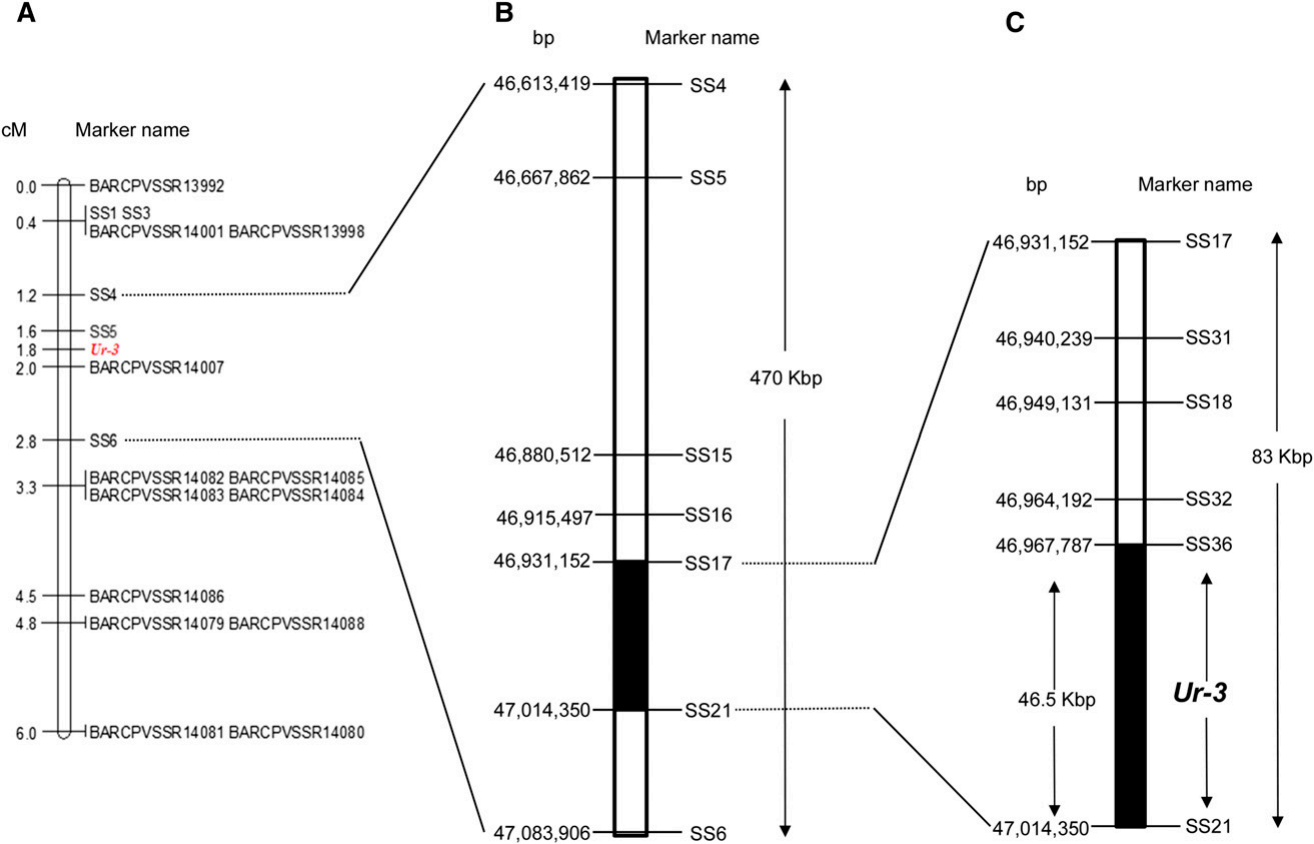
Genetic and physical map of the *Ur-3* locus in chromosome Pv11 of common bean. *Ur-3* confers resistance to the bean rust pathogen (*Uromyces appendiculatus*). (A) SSR (identified as BARCPVSSR) and SNP KASP markers (identified as SS) positioned the *Ur-3* locus between KASP marker SS5 and SSR marker BARCPVSSR14007 in a 470 kb genomic region in Pv11. (B) Flanking KASP markers SS4 and SS6 were used to genotype recombinant F_2:3_ families and to map the *Ur-3* locus in an 83-kb genomic region between KASP markers SS17 and SS21. (C) Haplotype analysis combined with genotyping of recombinant F_3_ plants positioned the *Ur-3* locus in a 46.5-kb genomic region between KASP markers SS36 and SS21. KASP marker SS68 is tightly linked to the *Ur-3* locus in this region. The genetic map was generated using the Kosambi mapping function from 129 F_2_ plants derived from Pinto 114 (susceptible) × Aurora cross (resistant with *Ur-3*).

The large portion of the genomic region containing the 28 SNPs associated with the *Ur-3* rust resistance gene was targeted for SSR development. A total of 48 SSR markers located between 46,266,888 and 48,664,905 bp on Pv11 were developed. Thirteen of the 48 SSRs markers were polymorphic between the parents Pinto 114 (susceptible) and Aurora (resistant) parents (Table 3). These markers, which showed unequivocal allele separation in agarose gel, were used to map the *Ur-3* locus in the F_2_ population Pinto 114 × Aurora. Linkage analysis positioned the *Ur-3* locus between markers BARCPVSSR14001 (46,535,562 bp) and BARCPVSSR14082 (47,291,606 bp), a 756,044 bp genomic region (data not shown). In addition, four positively associated SNPs from the BSA and two SNPs [retrieved from Song *et al.* (2015)] near the SSRs flanking the *Ur-3* locus were selected and converted into KASP markers (Table 4). Five KASP markers (SS1, SS3, SS4, SS5, and SS6) showed clear separation of the three clusters (two homozygous and one heterozygous) and were polymorphic between the Pinto 114 and Aurora parents. These KASP markers were used to refine the *Ur-3* gene map. Linkage analysis in the F_2_ population genotyped with 13 SSRs and the five KASP markers showed that *Ur-3* was flanked by KASP marker SS5 and SSR marker BARCPVSSR14007 between 46,667,862 and 46,865,194 bp, respectively, on chromosome Pv11 (Figure 1A and Table S3). The distance of the *Ur-3* locus to these flanking markers was 0.2 cM (Figure 1A).

**Table 3:** Simple sequence repeat (SSR) marker ID, motif, and forward and reverse primer position on version V1.0 of the reference genome of *Phaseolus vulgaris* and primer sequences

SSR BARC ID	Motif	Product Size	Forward Primer Position (bp)	Reverse Primer Position (bp)	Forward Primer Sequence (5′–3′)	Reverse Primer Sequence (5′–3′)
BARCPVSSR13992	(AT)10	293	46,266,888	46,267,182	CAAATCCTAAGTGTCATCGCAA	TTTCCCATCCATATCATTCCA
BARCPVSSR13998	(TA)10	280	46,402,850	46,403,129	TTGGTGATCGAAAGGTATCC	GGCTTTCTTTCCCTTTGTCC
BARCPVSSR14001	(TA)16	234	46,535,562	46,535,795	TCTGAATTTTATTTCAGTTGCTCC	TGTCTTGGGTTGAGATGTATGA
BARCPVSSR14007	(TC)12	276	46,865,194	46,865,469	CCTCTGATTTTTGGTCATGGA	AAGCAATGGAAATGCAAGATG
BARCPVSSR14082	(AT)17	206	47,291,401	47,291,606	TCTGAAATCATAGGCCAGCA	CCCACCTTTACATTTCCAACA
BARCPVSSR14083	(TA)10	282	47,336,615	47,336,896	TGATCATTTCTGCTATCATGGG	ATCACACTGCAACCACCAGA
BARCPVSSR14084	(AT)20	225	47,398,825	47,399,049	TGTCTTAATGTTGTGGGTGTGT	AATGCTCCCATCAAAACTCG
BARCPVSSR14085	(TTA)29	243	47,718,795	47,719,037	TGGATGACGTTCCACTCGTA	TTTTAACACATCACCCTCTTCTTT
BARCPVSSR14086	(AT)12	155	47,967,465	47,967,619	GGCCTCAGACTGGTGAGTGT	ACCATCCGAAAAGGGTTTCT
BARCPVSSR14088	(ATA)21	169	48,416,592	48,416,760	AAGGAACATAGCACATTTTACACA	CCAACACAAAATCGCTTTCA
BARCPVSSR14079	(TAT)10	171	48,441,279	48,441,449	CCAACTTTCTCACAGTCACATCA	TGCTTGACTAAGTCCTATGGAGA
BARCPVSSR14080	(ATT)13(TAT)10	279	48,565,882	48,566,160	CCATAAGTCTCACCCTGTTTTT	GCATAGCAGGCCTACCACA
BARCPVSSR14081	(TG)10	298	48,664,607	48,664,905	GCCGTACACTAAAGAAGGCA	CCTTTAAGGACCTTGTTTGGA

The SSRs were polymorphic between the parent Pinto 114 (susceptible) and Aurora (resistant with *Ur-3*) and were used to map the *Ur-3* rust resistance locus.

**Table 4:** Physical position and primer sequences of KASP markers associated with *Ur-3* rust resistance gene in common bean

Short Marker Name	Physical Position Pvul V1.0 (bp)	KASP Assay Primer Sequences (5′–3′)
KASP markers developed based on the bulk segregant analysis found in the BARC Bean6k_3 Beadchip and Song *et al.* (2015)		
SS1	46,437,627	GAAGGTGACCAAGTTCATGCTCACATGGCTGAGGAGGAGTAATTAT
		GAAGGTCGGAGTCAACGGATTACATGGCTGAGGAGGAGTAATTAG
		CTGCGGGTGCTTTGTATCATCAACAA
SS3	46,494,532	GAAGGTGACCAAGTTCATGCTAGGTTATAATACTTGGAGAACATGCAG
		GAAGGTCGGAGTCAACGGATTGAGGTTATAATACTTGGAGAACATGCAA
		GTTCTCCAGTATTCTCAACCTATGCAAAT
SS4	46,613,419	GAAGGTGACCAAGTTCATGCTCACACAGATCAATTACAGTGATACCA
		GAAGGTCGGAGTCAACGGATTCACACAGATCAATTACAGTGATACCC
		GACAACAATAGCTCACTGTGATGCCAT
SS5	46,667,862	GAAGGTGACCAAGTTCATGCTTGTTTCCTCAACCTGTGATTCTCC
		GAAGGTCGGAGTCAACGGATTTGTTTCCTCAACCTGTGATTCTCT
		TATCAGAAAAGATGGCCACTTTGTTTTGAA
SS6	47,083,906	GAAGGTGACCAAGTTCATGCTGGTAACTACAAGAGATACAAACCAAC
		GAAGGTCGGAGTCAACGGATTTGGTAACTACAAGAGATACAAACCAAA
		CCCCAACCTAAATGAAAAATTCTGACATAT
KASP markers for the genomic region delimited by SS4 and SS6 markers flanking *Ur-3* found in the whole genome sequencing project		
SS15	46,880,512	GAAGGTGACCAAGTTCATGCTCATGTTYAGCAAAAACTTGCCAACTATG
		GAAGGTCGGAGTCAACGGATTCATGTTYAGCAAAAACTTGCCAACTATA
		AAAGTTGCTACTACTATGCAGTCACATAAA
SS16	46,915,497	GAAGGTGACCAAGTTCATGCTTACTTTCATCCTTATTTTGCACCCTC
		GAAGGTCGGAGTCAACGGATTATATTACTTTCATCCTTATTTTGCACCCTA
		GTGTATATATATATACACATASATACACTA
SS17	46,931,152	GAAGGTGACCAAGTTCATGCTATGTCTAAGGGGTTTGTCCACAA
		GAAGGTCGGAGTCAACGGATTATGTCTAAGGGGTTTGTCCACAT
		CAGTCATGCAAAAAATACCATRCAGAAGAA
SS31	46,940,239	GAAGGTGACCAAGTTCATGCTGTGGTTGTAGATTTCAAACAATAAGATTTTG
		GAAGGTCGGAGTCAACGGATTGTGGTTGTAGATTTCAAACAATAAGATTTTC
		TAGCTACTTCACACAACTTATCTAAACCAT
SS18	46,949,131	GAAGGTGACCAAGTTCATGCTATATGASATGGTGCTGTGGACAAC
		GAAGGTCGGAGTCAACGGATTCATATGASATGGTGCTGTGGACAAT
		AAGAAAGGGTTCTGAAAATTGGAAGTGAA
SS32	46,964,192	GAAGGTGACCAAGTTCATGCTGAATAGGAATCAAGAAAGTTGAAAAACTC
		GAAGGTCGGAGTCAACGGATTCGAATAGGAATCAAGAAAGTTGAAAAACTT
		CAAAAGACAGATATCCCCTTCCAAGTATA
SS36	46,967,787	GAAGGTGACCAAGTTCATGCTCAAAAAAGCAGTTCTGCACATACAAATG
		GAAGGTCGGAGTCAACGGATTCAAAAAAGCAGTTCTGCACATACAAATA
		GTTTCTCAAGTCTCATGAAATTCACAGTTT
SS68	46,967,980	GAAGGTGACCAAGTTCATGCTTGTGAATGGTATAATATTAAACGACCTCA
		GAAGGTCGGAGTCAACGGATTGTGAATGGTATAATATTAAACGACCTCT
		AGTRCATTGGATTCAATGTCTTCAACA
SS19	46,971,604	GAAGGTGACCAAGTTCATGCTAAATTCAGAGCATTTTTTAATTGTCAGACC
		GAAGGTCGGAGTCAACGGATTCAAATTCAGAGCATTTTTTAATTGTCAGACT
		ACCTACAGATGATATCACAGGGGCA
SS20	47,000,518	GAAGGTGACCAAGTTCATGCTGATGGTCATCAAAGGTAGGTCGAT
		GAAGGTCGGAGTCAACGGATTATGGTCATCAAAGGTAGGTCGAC
		ACATCTCCAGTAGAAGATGAAATGGACTT
SS21	47,014,350	GAAGGTGACCAAGTTCATGCTGTTGAAAGAATCTTCGCACAGGAAAAA
		GAAGGTCGGAGTCAACGGATTGAAAGAATCTTCGCACAGGAAAAG
		AATAGTATTGAGTGTTGCTTGTTACAGTWT

KASP markers were used to genotype the F_2_ mapping population and F_3_ families for fine mapping from the cross Pinto 114 (susceptible) × Aurora (resistant with *Ur-3*).

### Analysis of recombination in F_3_ and Ur-3 haplotype identification

KASP markers SS4 and SS6 were mapped at 0.6 and 1.0 cM from the *Ur-3* locus, respectively (Figure 1A), in a 470,487 bp (470 kb) genomic region of chromosome Pv11, from 46,613,419 to 47,083,906 bp (Figure 1B). These markers were chosen to genotype 12 selected F_3_ families from the cross Pinto 114 × Aurora. Among the 12 families, four were derived from recombinant F_2_ plants between KASP markers SS4 and SS6, six families were heterozygous between markers SS4 and SS6 flanking *Ur-3*, and two families were used as internal controls: one homozygous resistant and the other homozygous susceptible. In addition, these 12 families (281 F_3_ plants) were inoculated with race 31-1 of *U. appendiculatus*. Genotyping the 281 F_3_ plants resulted in 87 F_3_ plants with recombination events between the SS4 and SS6 KASP markers (Table S4). These 87 F_3_ plants were selected for subsequent fine-mapping analysis with additional KASP markers (Table 4). SS5 (ss715647451 at position 46,667,862) was the only KASP marker derived from the BeanChip that was located between SS4 and SS6; thus, SS5 was also used to genotype the recombinant 87 F_3_ plants.

We then mined the SNP sequence data from the 18 common bean varieties (Song *et al.* 2015) to search for additional SNPs between SS4 and SS6. Based on the whole genome sequence of the 18 common bean varieties, ∼6000 SNPs and small indels were found between SS4 and SS6 (Table S5). These SNPs were grouped into 10 major haplotypes (Table 5). Each of these haplotypes were then tagged with one or two KASP markers and were examined for their polymorphism between Pinto 114 (*ur-3*), Aurora (*Ur-3*), Mexico 235 (*Ur-3+*), and PI 181996 (*Ur-11*). The KASP markers polymorphic between the Pinto 114 and Aurora parents were tested on the set of 87 F_3_ recombinant plants identified previously with KASP markers SS4 and SS6. Analysis of the 87 F_3_ recombinant plants positioned the *Ur-3* gene between KASP markers SS17 and SS21, in the 83,198 bp genomic region (Figure 1B and Table S7). Concurrently, a specific haplotype for *Ur-3* was identified based on the reaction of the 18 sequenced varieties to race 31-1 of *U. appendiculatus*. Only the varieties C 20, Matterhorn, Stampede, T-39, and Sierra had a resistant phenotype (HR) to races 31-1 and 22-52, indicating that these cultivars have the *Ur-3* gene (Table S7). The final genotyping analysis on the 87 recombinant plants mapped *Ur-3* between KASP markers SS36 and SS21, in a specific genomic region of 46,563 bp, ranging from 46,967,787 to 47,014,350 bp of Pv11 (Table 6). Two F_3_ plants, one resistant and the other susceptible, had the same recombination breakpoint, demonstrating that the *Ur-3* gene was located in the region flanked by SS36 and SS21 (Figure 1C and Table 6).

**Table 5:** Major haplotypes identified between KASP markers SS4 and SS6 using SNP calls from 18 sequenced common bean varieties (Song *et al.* 2015) and G19833, the common bean reference genome landrace.

Sequenced Bean Varieties	Market Class	Common bean race	Presence of *Ur-3*^Δ^	Marker names (from SS4 to SS6), their positions on chromosome Pv11, and the major haplotypes										
				SS4	SS5	SS15	SS16	SS17	SS68	SS18	SS19	SS20	SS21	SS6
				46613419	46667862	46880512	46915497	46931152	46967980	46949131	46971604	47000518	47014350	47083906
G19833 (Ref)	Landrace	Peru	S	T	C	C	C	T	A	C	G	T	T	C
Cal Early	Light Red Kidney	Nueva Granada	S	T	C	C	*	T	A	C	G	T	C	A
Red Hawk	Dark Red Kidney	Nueva Granada	S	T	C	C	A	T	A	C	G	T	C	A
Fiero	Dark Red Kidney	Nueva Granada	S	T	C	C	A	T	A	C	G	T	C	A
Lark	Light Red Kidney	Nueva Granada	S	T	C	C	A	T	A	C	G	T	C	A
Kardinal	Light Red Kidney	Nueva Granada	S	T	C	C	A	T	A	C	G	T	C	A
BAT 93	Tan	Mesoamerica	S	T	C	T	A	T	A	C	G	*	*	*
UC White	White Kidney	Nueva Granada	S	T	C	T	A	T	A	C	G	T	C	H
Jalo EEP 558	Canário	Peru	S	T	C	*	C	T	A	C	G	T	T	A
UI 906	Black	Mesoamerica	S	T	C	T	A	T	*	*	A	C	T	C
Michelite	Navy	Mesoamerica	S	G	H	C	C	T	A	T	A	C	T	C
Cornell 49242	Black	Mesoamerica	S	T	C	C	C	T	A	T	A	C	T	*
Laker	Navy	Mesoamerica	S	T	H	T	C	T	H	C	A	C	T	*
Buckskin	Pinto	Durango	S	T	C	T	C	T	H	T	A	C	C	C
T-39	Black	Mesoamerica	R	G	T	T	*	*	T	T	A	C	*	A
Sierra	Pinto	Durango	R	G	T	T	C	A	T	T	A	C	T	A
Matterhorn	Great Northern	Durango	R	H	C	T	C	A	T	*	A	C	T	A
Stampede	Pinto	Durango	R	G	C	T	C	A	T	T	A	C	T	A
C 20	Navy	Mesoamerica	R	H	H	T	C	A	T	T	A	C	T	A

The haplotype associated with the *Ur-3* bean rust resistance locus in common bean is revealed by markers SS17 and SS68 at positions 46,931,152 bp and 46,967,980 bp respectively. H = Heterozygous, *= missing data, S = Susceptible, R = Resistant, ^Δ^= based on HR (hypersensitive response) resistant reaction to race 31-1 of *Uromyces appendiculatus*.

**Table 6:** Genotypes (AA, BB) at nine SNP loci (from SS4 to SS6), and the reaction to race 31-1 of *Uromyces appendiculatus* of 87 F_3_ plants with recombination events from a Pinto 114 (susceptible) × Aurora (resistant with *Ur-3*) population.

		Nine KASP markers, their position on chromosome Pv11, and their genotypes								
		SS4	SS5	SS17	SS31	SS32	SS36	SS68	SS21	SS6
No F_3_ plants	Reaction to race 31-1	46613419	46667862	46931152	46940239	46964192	46967787	46967980	47014350	47083906
1 plant	Susceptible	AA	AA	AA	AA	AA	AA	AA	AA	AA
14 plants	Resistant	BB	BB	BB	BB	BB	BB	BB	BB	BB
1 plant	Susceptible	AA	AA	AA	AA	AA	AA	AA	AA	BB
**1 plant**	Susceptible	AA	AA	AA	AA	AA	AA	AA	BB	BB
9 plants	Resistant	AA	AA	BB	BB	BB	BB	BB	BB	BB
43 plants	Resistant	BB	BB	BB	BB	BB	BB	BB	BB	AA
**1 plant**	Resistant	BB	BB	BB	BB	BB	BB	BB	AA	AA
17 plants	Susceptible	BB	AA	AA	AA	AA	AA	AA	AA	AA

The two bolded F_3_ plants had the same recombination breakpoint but different phenotypes (AA = Pinto 114 allele; BB = Aurora allele) indicating the location of the *Ur-3* locus

**Figure 2:**
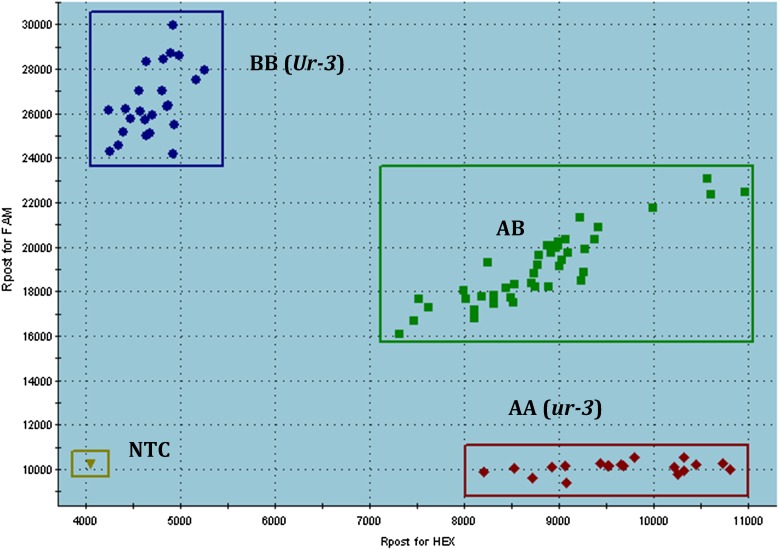
KASP marker SS68 analyzed on 129 F_2_ plants from the cross Pinto 114 (susceptible) × Aurora (resistant with the *U-3 locus*) cross inoculated with races of the bean rust pathogen (*Uromyces appendiculatus*). AA, *ur-3* alleles; AB, heterozygous alleles; BB, *Ur-3* alleles; NTC, nontarget control.

Subsequent genotyping of the 129 F_2_ plants from the Pinto 114 × Aurora cross using KASP SS36 and KASP marker SS68, which was targeting the *Ur-3* haplotype and only ∼200 bp downstream from SS36, showed that these markers were linked to the *Ur-3* rust resistance gene, with no recombination observed between bean rust phenotype and genotype (Table S2). SNP for KASP marker SS68 (46,967,980 bp in Pv11) is a transversion nucleotide change from A to T, where A is susceptible and T is resistant. KASP marker SS68 effectively differentiated homozygous-resistant, homozygous-susceptible, and heterozygous plants (Figure 2). Conversely, the KASP marker SS36 did not always differentiate homozygous-resistant from heterozygous plants (data not shown). KASP marker SS68 is located proximal (∼500 bp) to the leucine-rich repeat–containing gene, Phvul.011G193100.

### Validation of KASP marker SS68 linked to the Ur-3 gene

We used the SS68 KASP marker to genotype a panel of 130 common bean cultivars that included dry and snap beans. Some of these common beans possessed the *Ur-3* gene alone, while others had *Ur-3* in combination with other rust resistance genes. In addition, other cultivars had single or combinations of the other 10 rust resistance genes in common bean. The results of this validation showed that SS68 was highly accurate for the identification of the *Ur-3* locus (Table 7). No false positives or false negatives were observed when comparing the genotypic (evaluation with SS68 marker) and phenotypic (reaction to race 31-1) evaluations of these cultivars.

**Table 7:** Validation of the KASP marker SS68 tightly linked with the *Ur-3* rust resistance locus on 130 common bean cultivars

Genotype	*Ur* Gene*^a^*	Dry/Snap Bean	SS68*^b^*
Pinto 114	*ur-3*	Dry bean	AA
Aurora	*Ur-3*	Dry bean	BB
Mexico 235	*Ur-3+*	Dry bean	BB
Ecuador 299	*Ur-3+*	Dry bean	BB
NEP 2	*Ur-3+*	Dry bean	BB
51051	*Ur-3+*	Dry bean	BB
Early Gallatin	*Ur-4*	Snap bean	AA
Mexico 309	*Ur-5*	Dry bean	AA
Golden Gate Wax	*Ur-6*	Snap bean	AA
GN 1140	*Ur-7*	Dry bean	AA
PI 181996	*Ur-11*	Dry bean	AA
PC 50	*Ur-9*; *Ur-12*	Dry bean	AA
Redlands Pioneer	*Ur-13*	Dry bean	AA
Ouro Negro	*Ur-14*	Dry bean	AA
Condor	Susc; reported with *Ur-3*	Dry bean	AA
Vista	Susc; reported with *Ur-3*	Dry bean	AA
Raven	Susc; reported with *Ur-3*	Dry bean	AA
Jaguar	Susc; reported with *Ur-3*	Dry bean	AA
Santa Fe	*Ur-3*	Dry bean	BB
Merlot	*Ur-3*	Dry bean	BB
Stampede	*Ur-3*	Dry bean	BB
Alpine	*Ur-3*	Dry bean	BB
Starlight	*Ur-3*	Dry bean	BB
CO-54150	*Ur-3*	Dry bean	BB
C 20	*Ur-3*	Dry bean	BB
Matterhorn	*Ur-3*	Dry bean	BB
Chase	*Ur-3*	Dry bean	BB
Apache	*Ur-3*	Dry bean	BB
Burke	*Ur-3*	Dry bean	BB
La Paz	*Ur-3*	Dry bean	BB
Aztec	*Ur-3*	Dry bean	BB
T-39	*Ur-3*, *Ur-?*	Dry bean	BB
BelJersay-RR-1	*Ur-3*, *Ur-4*	Snap bean	BB
BelJersay-RR-4	*Ur-3*, *Ur-4*	Snap bean	BB
BelJersay-RR-5	*Ur-3*, *Ur-4*	Snap bean	BB
BelJersay-RR-6	*Ur-3*, *Ur-4*	Snap bean	BB
BelDade-RR-1	*Ur-3*, *Ur-4*	Snap bean	BB
BelDade-RR-2	*Ur-3*, *Ur-4*	Snap bean	BB
BelDade-RR-3	*Ur-3*, *Ur-4*	Snap bean	BB
BelDade-RGMR-4	*Ur-3*, *Ur-4*	Snap bean	BB
BelDade-RGMR-5	*Ur-3*, *Ur-4*	Snap bean	BB
Centennial	*Ur-3*, *Ur-6*	Dry bean	BB
Croissant	*Ur-3*, *Ur-6*	Dry bean	BB
CO-33875	*Ur-3*, *Ur-6*	Dry bean	BB
CO-34142	*Ur-3*, *Ur-6*	Dry bean	BB
CO-55119	*Ur-3*, *Ur-6*	Dry bean	BB
Kodiak	*Ur-3*, *Ur-6*	Dry bean	BB
Coyne	*Ur-3*, *Ur-6*	Dry bean	BB
ABC Weihing	*Ur-3*, *Ur-6*	Dry bean	BB
ABCP 8	*Ur-3*, *Ur-6*	Dry bean	BB
Stampede-R	*Ur-3*, *Ur-11*	Dry bean	BB
BelDak-RR-1	*Ur-3*, *Ur-6*, *CNC*	Dry bean	BB
BelDak-RR-2	*Ur-3*, *Ur-6*, *CNC*	Dry bean	BB
BelMiNeb-RMR-7	*Ur-3*, *Ur-4*, *Ur-11*	Dry bean	BB
BelDakMi-RMR-14	*Ur-3*, *Ur-6*, *Ur-11*	Dry bean	BB
BelDakMi-RMR-16	*Ur-3*, *Ur-6*, *Ur-11*	Dry bean	BB
BelDakMi-RMR-17	*Ur-3*, *Ur-6*, *Ur-11*	Dry bean	BB
BelDakMi-RMR-18	*Ur-3*, *Ur-4*, *Ur-6*, *Ur-11*	Dry bean	BB
BelMiNeb-RMR-8	*Ur-3*, *Ur-4*, *Ur-6*, *Ur-11*	Dry bean	BB
BelMiNeb-RMR-10	*Ur-3*, *Ur-4*, *Ur-6*, *Ur-11*	Dry bean	BB
BelMiNeb-RMR-11	*Ur-3*, *Ur-4*, *Ur-6*, *Ur-11*	Dry bean	BB
BelMiNeb-RMR-12	*Ur-3*, *Ur-4*, *Ur-6*, *Ur-11*	Dry bean	BB
Slenderette	*Ur-4*	Snap bean	AA
Caprice	*Ur-4*	Snap bean	AA
Gold Rush	*Ur-4*	Snap bean	AA
Acclaim	*Ur-4*	Snap bean	AA
B-190	*Ur-5*	Dry bean	AA
Olathe	*Ur-6+*	Dry bean	AA
BeldakMi-RR-4	*Ur-11*	Dry bean	AA
BelMiNeb-RR-2	*Ur-11*	Dry bean	AA
BelMidak-RR-3	*Ur-11*	Dry bean	AA
BelMidak-RR-4	*Ur-11*	Dry bean	AA
BARC-RR-3	*Ur-4*; *Ur-5*	Snap bean	AA
BARC-RR-17	*Ur-4*; *Ur-5*	Snap bean	AA
BARC-RR-18	*Ur-4*; *Ur-5*	Snap bean	AA
BARC-RR-24	*Ur-4*; *Ur-5*	Snap bean	AA
BARC-RR-25	*Ur-4*, *Ur-5*	Snap bean	AA
BARC-RR-26	*Ur-4*, *Ur-5*	Snap bean	AA
BARC-RR-27	*Ur-4*, *Ur-5*	Snap bean	AA
BelMiNeb-RR-1	*Ur-4*, *Ur-11*	Dry bean	AA
BelMiNeb-RMR-3	*Ur-4*, *Ur-11*	Dry bean	AA
BelMidak-RR-1	*Ur-4*, *Ur-11*	Dry bean	AA
BelMidak-RR-2	*Ur-4*, *Ur-11*	Dry bean	AA
BelJersey-RR-10	*Ur-4*, *Ur-11*	Snap bean	AA
BelJersey-RR-11	*Ur-4*, *Ur-11*	Snap bean	AA
BelJersey-RR-12	*Ur-4*, *Ur-11*	Snap bean	AA
BelJersey-RR-18	*Ur-4*, *Ur-11*	Snap bean	AA
BelFla-RR-3	*Ur-4*, *Ur-11*	Snap bean	AA
BelFla-RR-4	*Ur-4*, *Ur-11*	Snap bean	AA
BelTenn-RR-1	*Ur-4*, *Ur-11*	Snap bean	AA
BelTenn-RR-2	*Ur-4*, *Ur-11*	Snap bean	AA
BeltGlade-RR-2	*Ur-4*, *Ur-11*	Snap bean	AA
BeltGlade-RR-3	*Ur-4*, *Ur-11*	Snap bean	AA
Cabot	*Ur-4*, *Ur-11*	Snap bean	AA
Clarke	*Ur-4*, *Ur-11*	Snap bean	AA
Montrose	*Ur-5*, *Ur-7*	Dry bean	AA
Kimberly	*Ur-5*, *Ur-?*	Dry bean	AA
BelDakMi-RR-1	*Ur-6*, *Ur-11*	Dry bean	AA
BelDakMi-RR-2	*Ur-6*, *Ur-11*	Dry bean	AA
BelDakMi-RR-3	*Ur-6*, *Ur-11*	Dry bean	AA
BelDakMi-RR-5	*Ur-6*, *Ur-11*	Dry bean	AA
BelDakMi-RMR-13	*Ur-6*, *Ur-11*	Dry bean	AA
Buster	*Ur-3*, *Ur-5*, *Ur-7*	Dry bean	BB
BelMiNeb-RMR-4	*Ur-4*, *Ur-6*, *Ur-11*	Dry bean	AA
BelMiNeb-RMR-5	*Ur-4*, *Ur-6*, *Ur-11*	Dry bean	AA
BelMiNeb-RMR-6	*Ur-4*, *Ur-6*, *Ur-11*	Dry bean	AA
BelNeb-RR-1	*Ur-5*, *Ur-6*, *Ur-7*	Dry bean	AA
BelNeb-RR-2	*Ur-5*, *Ur-6*, *Ur-7*	Dry bean	AA
PI 151385	*Ur-11*	Dry bean	AA
PI 151388	*Ur-11*	Dry bean	AA
PI 151395	*Ur-11*	Dry bean	AA
PI 190078	*Ur-11*	Dry bean	AA
Zenith	*ur-3*	Dry bean	AA
Zorro	*ur-3*	Dry bean	AA
Amendoim Cavalo	*ur-3*	Dry bean	AA
G372	*ur-3*	Snap bean	AA
G1248	*ur-3*	Dry bean	AA
Volta	*ur-3*	Snap bean	AA
PV 718	*ur-3*	Snap bean	AA
Concessa	*ur-3*	Snap bean	AA
Crocket	*ur-3*	Snap bean	AA
Wyat	*ur-3*	Snap bean	AA
Harris	*ur-3*	Dry bean	AA
Neb#1 Sel	*ur-3*	Dry bean	AA
Beryl	*ur-3*	Dry bean	AA
Beryl-R	*ur-3*	Dry bean	AA
Pink Floyd	*ur-3*	Dry bean	AA
Bill-Z	*ur-3*	Dry bean	AA
Topaz	*ur-3*	Dry bean	AA

The marker was validated in a panel containing 130 Andean and Middle American common bean cultivars with and without *Ur-3*. These common beans represent most of the market classes planted in the United States. Susc, Susceptible based on phenotype reaction to race 31-1 of *Uromyces* appendiculatus; *Ur-?*, unknown rust resistant gene; CNC, Compuesto Negro de Chimaltenango.

a Ur gene identified based on phenotypic characterization using multiple races of *U. appendiculatus*.

b Allele score generated by KASP marker SS68 described in this study.

### The Ur-3 locus contains six candidate genes

The genomic region delimited by markers SS36 and SS21, defined as the *Ur-3* locus, contained six candidate genes according to the Phytozome.net database for *P. vulgaris* assembly V1.0. The names of these genes are: Phvul.011G193100, Phvul.011G193200, Phvul.011G193300, Phvul.011G193400, Phvul.011G193500, and Phvul.011G193600. Three of these *Ur-3* genes (Phvul.011G193100, Phvul.011G193500, and Phvul.011G193600) are classified as containing NB-ARC domains and leucine-rich repeat (LRR) regions. Genes Phvul.011G193200 and Phvul.011G193400 are annotated as serine/threonine kinases, and Phvul.011G193300 is a tyrosine kinase with salt/stress response–related and antifungal function. All these candidate genes, except Phvul.011G193600, were highly expressed in common bean leaves, according to the expression level experiments recorded in the JGI genome browser for *P. vulgaris*.

## Discussion

### Development of accurate SNP markers linked to the Ur-3 locus

The historically important *Ur-3* gene confers resistance to the pathogen that causes the rust disease of common bean. The effective incorporation of *Ur-3* into dry and snap beans using molecular markers has been limited by the inaccuracy of the molecular markers linked to this gene (Haley *et al.* 1994; Nemchinova and Stavely 1998; Stavely 2000). The authors that reported the RAPD (OK14_620_) and SCAR (SK14) markers linked to *Ur-3* indicated that these markers produced both false negatives and false positives results (Haley *et al.* 1994; Nemchinova and Stavely 1998).

More recently, we have used BSA, SNP assay, and whole genome sequencing to discover SSR markers closely linked to the *Ur-3* and other disease resistance genes. However, even the use of closely linked BARCPVSSR14007, an SSR marker reported in this study positioned at 0.2 cM from the *Ur-3* locus, resulted in >3% false positive results when this marker was used on the panel of 130 common bean lines (data not shown). Additionally, as indicated earlier, the inability to find specific molecular markers linked to *Ur-3* may have been exacerbated by the presence of the *Ur-11* rust resistance gene that is closely linked to *Ur-3* on the terminal position of chromosome Pv11. Currently, the most reliable method to monitor for the presence of the *Ur-3* gene in dry and snap bean cultivars continues to be race 31-1 (53) of *U. appendiculatus*. Race 31-1 is used as a phenotypic marker that effectively identifies common bean plants with *Ur-3* alone or in combination (Pastor-Corrales 2002). However, phenotypic evaluations under greenhouse conditions are very laborious and time consuming (∼21 d). Moreover, due mostly but not only to the biotrophic condition of the rust pathogen, most breeders of dry and snap beans do not have the option of using this methodology.

Given the importance of *Ur-3*, we determined to search for highly accurate molecular markers linked to *Ur-3* using a fine-mapping approach. We employed a variety of technologies that included phenotyping with specific races of the bean trust pathogen, BSA coupled with high-throughput SNP genotyping using the BARCBEAN6K_3 BeadChip, SSR and KASP marker development, and local association analysis using SNPs from previous whole genome shotgun sequencing efforts. In summary, the combination of these technologies permitted the identification of KASP marker SS68, which was highly accurate in identifying the presence of *Ur-3* in a panel of 130 common bean cultivars that included dry and snap beans with and without the *Ur-3* gene. Marker SS68 was also tested on a mapping population of 184 F_2_ genotypes from the cross between Pinto 114 × Mexico 235 (*Ur-3*+). No recombination was observed between phenotype and the genotype in this study (data not shown). These results confirm the accuracy and utility of the KASP marker SS68 even when this marker is used on mapping populations in which the origin of the *Ur-3* gene is not the cultivar Aurora.

### Survey of the SS68 KASP marker in a common bean diversity panel

In this study, we determined the potential utility of the KASP SNP marker SS68 in a panel of common bean cultivars carrying different rust resistance genes and in bean lines representing the major common bean market classes in the United States. Marker SS68 reliably identified cultivars containing *Ur-3*, independent of the gene pool (Andean or Middle American), type of common bean (dry or snap), or market class of dry edible beans (pinto, great northern, navy, red kidney, black, and others). Additionally, SS68 effectively distinguished common bean lines carrying *Ur-3* alone as well as lines combining the *Ur-3* and *Ur-11* genes that are closely linked on Pv11 (Table 7). Because *Ur-3* gene is epistatic to *Ur-11*, it is difficult to combine these two genes using inoculations with races of the rust pathogen (Stavely 2000). Thus, using marker SS68 to identify *Ur-3* when combined with *Ur-11* avoids this problem.

### The Ur-3 locus maps to a 46 kb region possessing candidate genes with resistant gene motifs

Through haplotype analysis and KASP marker development, it was possible to determine a genomic region of 46,563 bp containing the *Ur-3* locus and delimited by markers SS36 and SS21 on Pv11. Six candidate genes were identified within this 46.5 kb region in the *P. vulgaris* reference genome, obtained by sequencing the landrace G 19833 of Andean origin. Among the six candidate genes, there were three genes with NB-ARC LRR domains. Proteins containing NB-ABC LRR domains are known to be involved in plant resistance and activation of innate immune responses to various types of pathogens (Hammond-Kosack and Jones 1997; Jones and Dangl 2006). Similarly, protein kinases (also found in the 46.5 kb region) are known to play a central role in signaling during pathogen recognition and the subsequent activation of plant defense mechanisms (Xue *et al.* 2015). The genomic region containing the *Co-4* gene on chromosome Pv08, conferring resistance to *Colletotrichum lindemuthianum* in common bean, has been characterized and known to contain an open reading frame coding for a serine threonine kinase (Oblessuc *et al.* 2015), a type of protein which has also been identified in our studies. Additionally, serine threonine protein kinase constitutes candidate genes for controlling angular leaf spot resistance in the Andean landrace G 5686 (Keller *et al.* 2015). Whether the phenotype of the *Ur-3* locus is the result of the expression of one or more of the six candidate genes will be a matter of further investigation.

Sequence analysis of the Andean landrace G 19833, used to sequence the reference genome of common bean, revealed that the 46.5 kb genomic region containing the *Ur-3* locus is highly duplicated (Figure S1), and it includes repetitive elements in the intergenic spaces. Additionally, this genomic region is AT-rich (33% *vs.* 16% for GC), which suggests that it is highly unstable. Sequence analysis comparing the Middle American Aurora common bean and the Andean landrace G 19833, will provide valuable insights into the structural differences and evolutionary history of the important *Ur-3* rust resistance locus.

### Conclusions

This study used a new approach to generate KASP SS68, the first highly accurate DNA marker linked to the *Ur-3* rust resistance gene in common bean. We fine-mapped a 46.5 kb genomic region in chromosome Pv11, present in Middle American common bean cultivar Aurora. This was accomplished using the BARCBEAN6K_3 BeadChip, SSRs, KASP technology, and local association. The validation of this newly discovered KASP SS68 marker on a panel of 130 common bean lines revealed that SS68 was highly accurate in identifying *Ur-3*. This marker will be of value for combining *Ur-3* with other Andean and Middle American genes with broad spectrum resistance to the highly variable bean rust pathogen. In addition, the utilization of the new marker SS68 will significantly reduce the time and labor associated with the transfer of the *Ur-3* gene using inoculations of bean plants with specific races of the rust pathogen. The genomic region containing the *Ur-3* locus included six genes annotated in the reference genome of *P. vulgaris*. These genes are likely candidates for the *Ur-3* rust resistance gene. Gene expression analysis of these candidate genes and functional approaches will enhance our understanding of the mechanisms underlying the reaction of *P. vulgaris* to *U. appendiculatus*.

## Supplementary Material

Supplemental material is available online at www.g3journal.org/lookup/suppl/doi:10.1534/g3.116.036061/-/DC1.
