# Supplementary material for: Fine Mapping of Ur-3, a Historically Important Rust Resistance Locus in Common Bean
Source: G3 (Bethesda). 2016 Dec 27;7(2):557–69. doi: 10.1534/g3.116.036061 (PMC5295601; doi:10.1534/g3.116.036061)
Supplement: Supplementary file 1 [file 557FigureS1.docx]

**Supplementary Information**


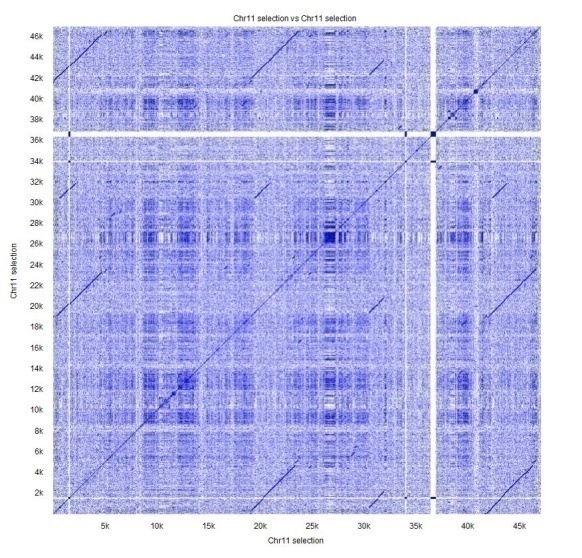


**Figure S1.** Dot plot comparison of the *Ur-3* locus comprising the 46.5 kbp region identified through fine mapping in this study. Sequence used for the analysis is from the reference genome G19833. Small solid lines above or below the diagonal line suggest the presence of duplicated or highly similar sequences within the region. Analysis done using CLC Genomics Workbench following manual instructions.
