# Supplementary material for: Fine Mapping of Ur-3, a Historically Important Rust Resistance Locus in Common Bean
Source: G3 (Bethesda). 2016 Dec 27;7(2):557–69. doi: 10.1534/g3.116.036061 (PMC5295601; doi:10.1534/g3.116.036061)
Supplement: Supplementary file 2 [file 557TableS1.docx]

Table S1. Reaction of bean rust differential cultivars to races of the bean pathogen used or named in the fine mapping of the Ur-3 rust resistance gene of common bean. (.xlsx, 11 KB)

<http://www.g3journal.org/lookup/suppl/doi:10.1534/g3.116.036061/-/DC1/TableS1.xlsx>
