# Supplementary material for: Fine Mapping of Ur-3, a Historically Important Rust Resistance Locus in Common Bean
Source: G3 (Bethesda). 2016 Dec 27;7(2):557–69. doi: 10.1534/g3.116.036061 (PMC5295601; doi:10.1534/g3.116.036061)
Supplement: Supplementary file 4 [file 557TableS2.docx]

Table S2. Rust phenotype and SS68 genotype of 129 F2 plants from cross Pinto 114 (S) x Aurora (R) to four races of *Uromyces appendiculatus*. (.xlsx, 18 KB)

<http://www.g3journal.org/lookup/suppl/doi:10.1534/g3.116.036061/-/DC1/TableS2.xlsx>
