# Supplementary material for: Fine Mapping of Ur-3, a Historically Important Rust Resistance Locus in Common Bean
Source: G3 (Bethesda). 2016 Dec 27;7(2):557–69. doi: 10.1534/g3.116.036061 (PMC5295601; doi:10.1534/g3.116.036061)
Supplement: Supplementary file 6 [file 557TableS3.docx]

Table S3. Genotype and rust phenotype of 129 F2 plants used for genetic linkage analysis around the Ur-3 locus for resistance to bean rust. (.xlsx, 24 KB)

[http://www.g3journal.org/lookup/suppl/doi:10.1534/g3.116.036061/-/DC1/TableS3.xlsx](http://www.g3journal.org/lookup/suppl/doi:10.1534/g3.116.036061/-/DC1/TableS2.xlsx)
