# Supplementary material for: Fine Mapping of Ur-3, a Historically Important Rust Resistance Locus in Common Bean
Source: G3 (Bethesda). 2016 Dec 27;7(2):557–69. doi: 10.1534/g3.116.036061 (PMC5295601; doi:10.1534/g3.116.036061)
Supplement: Supplementary file 10 [file 557TableS5.docx]

Table S5. SNPs and small indels used for haplotype analysis around the Ur-3 locus for resistance to bean rust. (.xlsx, 467 KB)

[http://www.g3journal.org/lookup/suppl/doi:10.1534/g3.116.036061/-/DC1/TableS5.xlsx](http://www.g3journal.org/lookup/suppl/doi:10.1534/g3.116.036061/-/DC1/TableS4.xlsx)
