# Supplementary material for: Fine Mapping of Ur-3, a Historically Important Rust Resistance Locus in Common Bean
Source: G3 (Bethesda). 2016 Dec 27;7(2):557–69. doi: 10.1534/g3.116.036061 (PMC5295601; doi:10.1534/g3.116.036061)
Supplement: Supplementary file 12 [file 557TableS6.docx]

Table S6. Genotype and rust phenotypes of 87 F3 recombinant plants used to fine mapped the Ur-3 locus. (.xlsx, 20 KB)

[http://www.g3journal.org/lookup/suppl/doi:10.1534/g3.116.036061/-/DC1/TableS6.xlsx](http://www.g3journal.org/lookup/suppl/doi:10.1534/g3.116.036061/-/DC1/TableS5.xlsx)
