# Supplementary material for: Fine Mapping of Ur-3, a Historically Important Rust Resistance Locus in Common Bean
Source: G3 (Bethesda). 2016 Dec 27;7(2):557–69. doi: 10.1534/g3.116.036061 (PMC5295601; doi:10.1534/g3.116.036061)
Supplement: Supplementary file 14 [file 557TableS7.docx]

Table S7. Rust phenotype of eighteen sequenced common bean lines with races 22-6, 31-1, 31-22, and 22-52 of *Uromyces appendiculatus*. (.xlsx, 16 KB)

<http://www.g3journal.org/lookup/suppl/doi:10.1534/g3.116.036061/-/DC1/TableS7.xlsx>
